# Comparative Study on the Effects of Silicon Nanoparticles and Cellulose Nanocrystals on Drought Tolerance in Tall Fescue (*Festuca arundinacea* Schreb.)

**DOI:** 10.3390/plants14101461

**Published:** 2025-05-14

**Authors:** Meng Li, Sile Hu, Xulong Bai, Jie Ren, Kanliang Tian, Huili Zhang, Zhilong Zhang, Vanquy Nguyen

**Affiliations:** 1 College of Soil and Water Conservation Science and Engineering, Northwest A&F University, Yangling 712100, China; limeng971207@163.com (M.L.); husilenwsuaf@163.com (S.H.); bxlnwafu@163.com (X.B.); 13150103148@163.com (J.R.); 2 College of Water Resources and Architectural Engineering, Northwest A&F University, Yangling 712100, China; 3 College of Forestry, Northwest A&F University, Yangling 712100, China; zhangzl@nwafu.edu.cn; 4 Southern Branch of Joint Vietnam-Russia Tropical Science and Technology Research Center, Hochiminh 740500, Vietnam; quynguyenvan45@gmail.com

**Keywords:** tall fescue, slope, drought stress, silicon nanoparticles, cellulose nanocrystals, transcriptome

## Abstract

Tall fescue (*Festuca arundinacea* Schreb.) is a herbaceous species that is commonly used for ecological slope restoration in China. However, water scarcity often constrains its growth due to the unique site conditions of steep slopes and climate-induced drought stress. This study aims to compare the ameliorative effects of silicon nanoparticles (Si NPs) and cellulose nanocrystals (CNCs) on drought stress in tall fescue and to elucidate their underlying mechanisms of action. The results indicated that drought stress impaired photosynthesis, restricted nutrient absorption, and increased oxidative stress, ultimately reducing biomass. However, Si NPs and CNCs enhanced drought tolerance and promoted biomass accumulation by improving photosynthesis, osmotic regulation, and antioxidant defense mechanisms. Specifically, Si NP treatment increased biomass by 48.71% compared to drought-stressed control plants, while CNCs resulted in a 33.41% increase. Transcriptome sequencing further revealed that both nanomaterials enhanced drought tolerance by upregulating genes associated with photosynthesis and antioxidant defense. Additionally, Si NPs improved drought tolerance by stimulating root growth, enhancing nutrient uptake, and improving leaf structure. In contrast, CNCs play a distinct role by regulating the expression of genes related to cell wall synthesis and metabolism. These findings highlight the crucial roles of these two nanomaterials in plant stress protection and offer a sustainable strategy for the maintenance and management of slope vegetation.

## 1. Introduction

The rapid expansion of infrastructure, including highways, railways, and water conservancy projects, has severely impacted local ecosystems in China. Large-scale engineering construction often leads to the formation of extensive exposed soil and rock slopes [1,2], resulting in soil degradation and increased risks of geological disasters such as debris flows, landslides, and collapses [3,4]. Various vegetation-based approaches for long-term erosion control have been employed to address these challenges, including external soil spray seeding, sprayed vegetation concrete, and vegetative blankets. These methods have played a crucial role in ecological restoration, soil consolidation, and water conservation [5,6]. However, their application in arid and semi-arid regions remains highly challenging. Limited precipitation, coupled with the shallow thickness of these artificially constructed soil layers, severely restricts water cycling. Over time, vegetation in these areas often experiences growth retardation, degradation, or even mortality due to drought-induced ecological imbalance. This phenomenon is particularly evident on steep slopes with an inclination angle greater than 45°, where vegetation degradation typically occurs within a year [7]. More critically, drought event duration, frequency, and spatial extent have recently increased [8]. This trend poses significant challenges to the restoration and sustainability of slope ecosystems in drought-affected, engineering-disturbed areas.

In arid environments, permanent irrigation systems and chemical agents are commonly used to mitigate drought stress. However, these measures fail to promote the self-repair capacity of ecosystems fundamentally. Long-term reliance on permanent irrigation systems imposes additional economic burdens, while chemical agents can pollute soil and water bodies, posing threats to environmental health [9,10]. Therefore, exploring a green and sustainable strategy to enhance the drought tolerance of slope vegetation has become an urgent research priority. Against this backdrop, the rapid advancement of nanotechnology offers new insights into improving the stress tolerance of slope vegetation. Various nanomaterials have recently been employed to enhance plant growth, productivity, and stress resilience [11]. Particularly, silicon nanoparticles (Si NPs) and cellulose nanocrystals (CNCs), owing to their biocompatibility, environmental friendliness, and unique advantages in endogenous physiological regulation (Si NPs) and exogenous water retention optimization (CNCs), provide an ideal research model for enhancing drought tolerance in slope plants [12,13]. Specifically, Si NPs directly improve metabolic homeostasis under drought conditions by activating key photosynthetic enzymes and promoting the accumulation of osmolytes such as soluble sugars, soluble proteins, and proline [14,15]. Furthermore, Si NPs significantly enhance plant drought tolerance by activating antioxidant defense systems (e.g., superoxide dismutase and peroxidase activity), reducing membrane lipid peroxidation damage, and synergistically regulating stomatal movement and cell wall stability, thereby forming a multi-dimensional synergistic physiological defense mechanism, as evidenced in wheat (*Triticum aestivum* L.) and rice (*Oryza sativa* L.) [16,17]. In contrast, CNCs, as emerging bio-based nanomaterials that can be synthesized or extracted from biological sources [18], have emerged as another potential solution for enhancing the drought tolerance in slope-protecting plants due to their unique swelling capacity and water retention properties [19]. Although few studies have investigated this role, a recent study reported that CNCs significantly improved leaf water retention by 200–300%, primarily due to increased leaf surface viscosity and enhanced wettability [20].

Tall fescue *(Festuca arundinacea* Schreb.), a cool-season grass species belonging to the Poaceae family, is widely used for slope ecological restoration in China. However, under drought conditions, it is prone to leaf yellowing, wilting, photosynthetic impairment, and reduced root activity, which can compromise ecological restoration outcomes. While both Si NPs and CNCs hold promise for enhancing plant drought tolerance, their applications in slope vegetation remain insufficiently explored, with the underlying mechanisms of CNCs being particularly unclear. Therefore, we will compare the effects of applying two exogenous nanomaterials and evaluate their impacts on the growth, photosynthesis, antioxidant enzyme activity, and nutrient uptake of tall fescue. Furthermore, RNA-Seq analysis will elucidate the molecular mechanisms by which these nanomaterials regulate plant drought tolerance. The findings of this study will provide new theoretical insights and technical support for the sustainable management of slope vegetation and promote the application of nanomaterials in the field of plant drought tolerance.

## 2. Results

### 2.1. Characteristics of Nanomaterials

**Figure 1:**
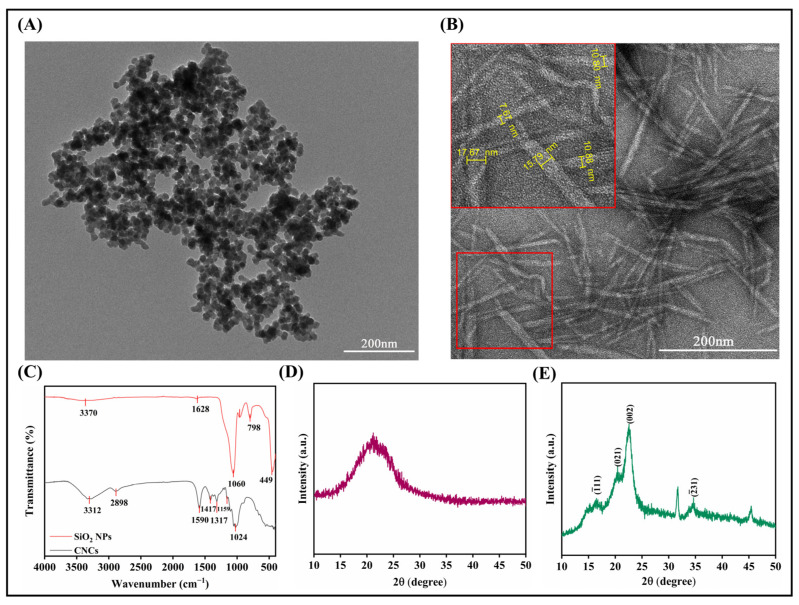
Structure and chemical characterization of two nanomaterials: (**A**) transmission electron microscope (TEM) image of Si NPs, (**B**) TEM image of CNCs, (**C**) Fourier transform infrared spectroscopy (FTIR) comparison of Si NPs and CNCs, (**D**) X-ray diffraction (XRD) pattern of Si NPs, and (**E**) XRD pattern of CNCs.

Transmission electron microscopy (TEM) analysis revealed that the silicon nanoparticles (Si NPs) exhibited a monodisperse solid spherical morphology with an average diameter of 20.09 ± 2.79 nm and a surface zeta potential of −28.27 ± 1.58 mV (Figure 1A and Figure S1A,C). In contrast, the cellulose nanocrystals (CNCs) displayed well-defined rod-like structures with an average length of 87.98 ± 7.17 nm and exhibited a stronger surface negative charge (zeta potential: −37.77 ± 1.31 mV) (Figure 1B and Figure S1B,D). Fourier-transform infrared (FTIR) spectroscopy (Figure 1C) further confirmed their distinct chemical characteristics: For Si NPs, the characteristic peaks at 3370 cm^−1^ and 1628 cm^−1^ corresponded to the O-H stretching vibration and bending vibration of physically adsorbed water on the surface, respectively. The strong absorption peak at 1060 cm^−1^ was attributed to the asymmetric stretching vibration of Si-O-Si bonds, while the weak peak at 963 cm^−1^ originated from the bending vibration of Si-OH. The symmetric stretching vibrations of Si-O at 798 cm^−1^ and 449 cm^−1^ collectively confirmed their amorphous siloxane structure. In comparison, the FTIR spectrum of CNCs showed a broad O-H stretching vibration peak at 3312 cm^−1^, which not only reflected adsorbed water but primarily indicated the strong hydrogen-bonding network formed by intermolecular and surface hydroxyl groups of cellulose. This high-density hydroxyl network immobilizes water molecules and forms a stable hydrated structure, thereby significantly enhancing water retention capacity. Additional characteristic peaks at 2898 cm^−1^ (C-H stretching vibration), 1024 cm^−1^ (C-O-C skeletal vibration of glycosidic rings), and 1590/1417 cm^−1^ (C-H in-plane bending and -CH_2_-deformation vibrations) further confirmed the polysaccharide structural features of cellulose.

X-ray diffraction (XRD) analysis demonstrated that the Si NPs exhibited a broad diffuse peak within 2θ = 15°–28° without detectable crystalline diffraction peaks of quartz or cristobalite (Figure 1D), confirming their typical amorphous structure. This broad peak correlated with the strong absorption at 1060 cm^−1^ in FTIR, jointly supporting the short-range ordered Si-O-Si network. The amorphous structure of Si NPs contributes to their relatively high specific surface area, which in turn facilitates the adsorption of water molecules. In contrast, the CNCs displayed characteristic diffraction peaks at 2θ = 16.449°, 20.606°, 22.572°, and 34.593° (Figure 1E), corresponding to the ($\bar{1}$11), (002), (021), and ($\bar{2}$31) crystal planes of cellulose I, respectively. These peaks indicate the preservation of the highly ordered crystalline architecture of natural cellulose I, consistent with the crystallographic features of cotton fibers.

### 2.2. Effects on Plant Growth

**Figure 2:**
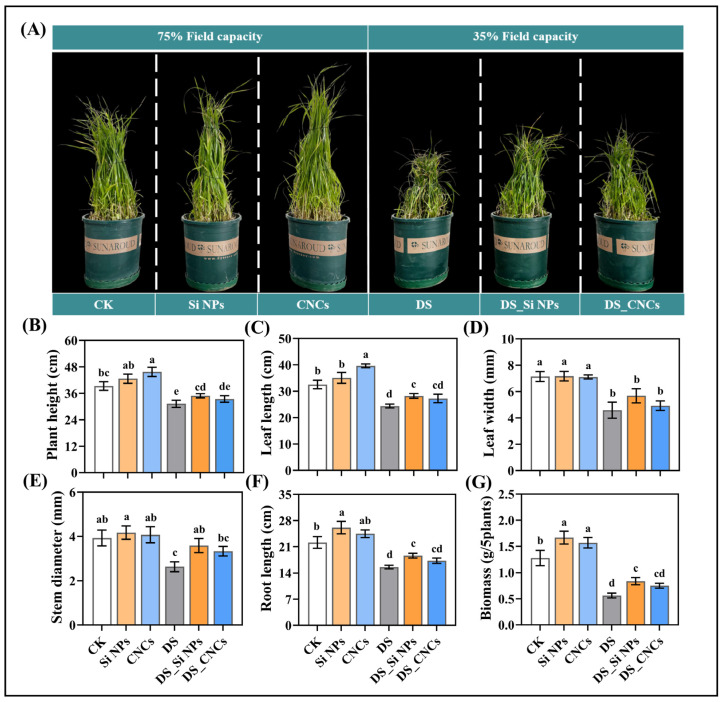
Effects of Si NP and CNC application on the growth of tall fescue under drought stress: (**A**) phenotypic comparison of different treatment groups, (**B**) plant height, (**C**) leaf length, (**D**) leaf width, (**E**) stem diameter, (**F**) root length, and (**G**) biomass. Data are presented as mean ± standard deviation from three biological experiments. Different letters above the bars indicate significant differences among treatments based on Tukey’s HSD test (one-way ANOVA, *p* < 0.05). Treatment abbreviations: CK (control, well-watered), Si NPs (well-watered + 300 mg/L silicon nanoparticles), CNCs (well-watered + 100 mg/L cellulose nanocrystals), DS (drought stress), DS_Si NPs (drought stress + 300 mg/L silicon nanoparticles), DS_CNCs (drought stress + 100 mg/L cellulose nanocrystals).

Phenotypic changes in plants treated with Si NPs and CNCs under both normal and drought conditions demonstrated the positive role of these nanomaterials in enhancing tall fescue growth and alleviating drought stress. Under normal conditions, plants treated with both nanomaterials exhibited greater growth vigor (Figure 2A). Specifically, Si NP treatment significantly promoted root growth and biomass accumulation in plants, with increases of 17.89% and 30.65% compared to control (CK) plants (*p* < 0.05), respectively. Interestingly, CNCs significantly enhanced plant height and leaf length, achieving increases of 16.38% and 21.65% (*p* < 0.05) (Figure 2B). Under drought stress, the control plants (DS) showed restricted growth and leaf wilting, particularly with biomass significantly reduced by 55.93% compared to CK plants (Figure 2A,B). However, both Si NPs and CNCs effectively alleviated drought-induced growth inhibition. Notably, Si NPs significantly improved stem diameter, root length, and biomass, which increased by 36.38%, 19.69%, and 48.71%, respectively, compared to DS plants (*p* < 0.05). Although CNC treatment also showed a certain degree of improvement, the differences in these parameters were not statistically significant compared to DS plants (*p* > 0.05) (Figure 2B). Overall, Si NPs exhibited a more pronounced positive effect on tall fescue growth under drought conditions, particularly in enhancing biomass accumulation.

### 2.3. Effects on Photosynthesis

**Figure 3:**
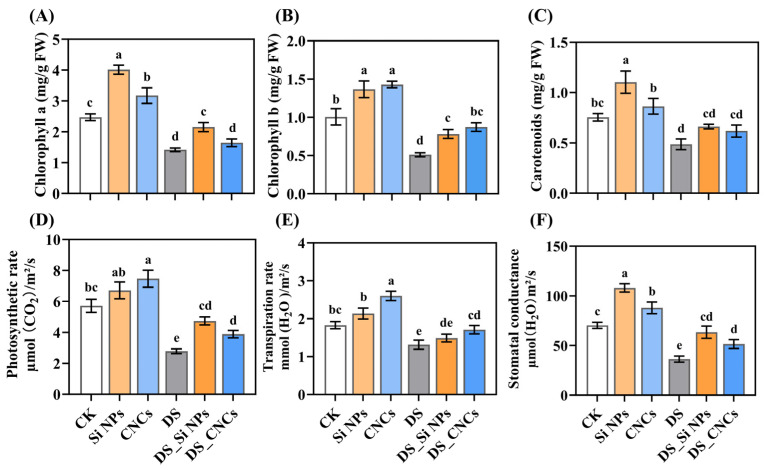
Effects of Si NPs and CNCs on photosynthetic pigment content and gas exchange parameters in tall fescue leaves under drought stress: (**A**) chlorophyll a, (**B**) chlorophyll b, (**C**) carotenoids, (**D**) photosynthetic rate, (**E**) transpiration rate, (**F**) stomatal conductance. Data are presented as means ± standard deviation from three biological experiments. Different letters above the bars indicate significant differences among treatments based on Tukey’s HSD test (one-way ANOVA, *p* < 0.05). Treatment abbreviations: CK (control, well-watered), Si NPs (well-watered + 300 mg/L silicon nanoparticles), CNCs (well-watered + 100 mg/L cellulose nanocrystals), DS (drought stress), DS_Si NPs (drought stress + 300 mg/L silicon nanoparticles), DS_CNCs (drought stress + 100 mg/L cellulose nanocrystals).

Figure 3 illustrates the effects of Si NPs and CNCs on photosynthetic pigment content and gas exchange parameters of tall fescue. Under normal conditions, the highest chlorophyll a and carotenoid contents were observed in Si NP plants, which increased by 62.31% and 46.33%, respectively, compared to control (CK) plants (*p* < 0.05) (Figure 3A,C). At the same time, the most pronounced improvement in stomatal conductance was also achieved (Figure 3F). In contrast, CNCs exhibited specific promoting effects on chlorophyll b content, photosynthetic rate, and transpiration rate, showing increases of 70.75%, 30.64%, and 42.16%, respectively, compared to CK plants (Figure 3B,D,E). Drought stress (DS) significantly suppressed photosynthesis and stomatal function, particularly reducing chlorophyll b content and photosynthetic rate by 49.29% and 51.25%, respectively, compared to CK plants (*p* < 0.05) (Figure 3B,D). However, both Si NPs and CNCs effectively alleviated the aforementioned negative effects. Specifically, compared to DS plants, Si NP treatment significantly increased chlorophyll a (51.65%) and chlorophyll b (53.28%) content, as well as photosynthetic rate (70.14%) and stomatal conductance (74.23%) (*p* < 0.05). Meanwhile, CNCs significantly enhanced chlorophyll b content, photosynthetic rate, transpiration rate, and stomatal conductance, increasing them by 70.75%, 39.44%, 30.14%, and 41.49%, respectively, compared to DS plants (Figure 3A–F).

### 2.4. Effects on Leaf Anatomical Structure

**Figure 4:**
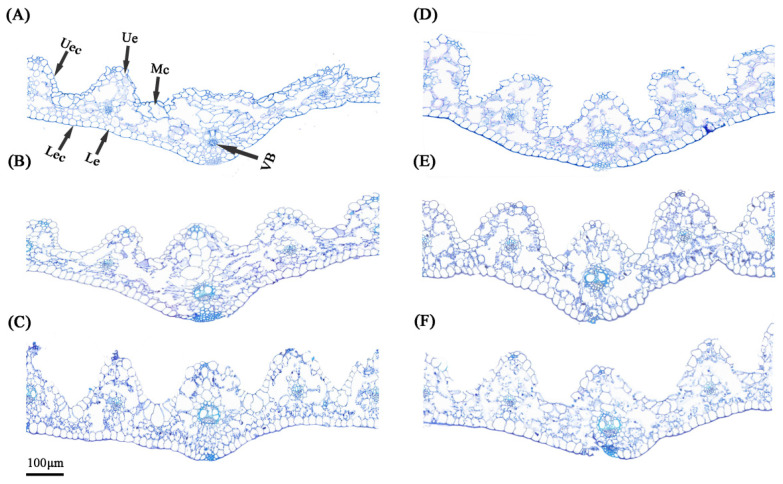
Anatomical images of tall fescue leaves treated with Si NPs and CNCs under drought stress: (**A**) CK, (**B**) Si NPs, (**C**) CNCs, (**D**) DS, (**E**) DS_Si NPs, (**F**) DS_CNCs.

The transverse section of tall fescue leaves consists of the epidermis, mesophyll, and vascular bundles. The epidermis comprises a single layer of nearly circular cells, with a thinner cuticle on the upper epidermis and a thicker cuticle on the lower epidermis. The mesophyll lacks distinct palisade and spongy tissues, forming an isolateral leaf structure. Additionally, multiple fan-shaped motor cells are distributed in the depressions between vascular bundles (Figure 4). Statistical analysis of various parameters (Table 1) revealed that, under normal conditions, Si NPs increased leaf thickness, upper epidermal cuticle thickness, and lower epidermal cuticle thickness compared to control (CK) plants (*p* < 0.05). Interestingly, CNC treatment significantly increased vascular bundle area by 97.83% (*p* < 0.05). Under drought stress, motor cells lose water, leading to leaf curling and structural changes in the leaves (Figure 4D). Drought stress resulted in a decrease in leaf thickness but an increase in other parameters, such as cuticle thickness, epidermal thickness, and vascular bundle area. The application of Si NPs further enhanced leaf thickness and other structural parameters under drought stress, showing significant differences from DS plants (*p* < 0.05), particularly in lower epidermal cuticle thickness, which increased by 86.81%. However, no significant effects of CNCs on leaf structural parameters were observed (*p* > 0.05), although a general improvement trend was present (Table 1).

### 2.5. Effects on MDA, Osmotic Adjustment Substances, and Antioxidant Enzyme Activity

**Figure 5:**
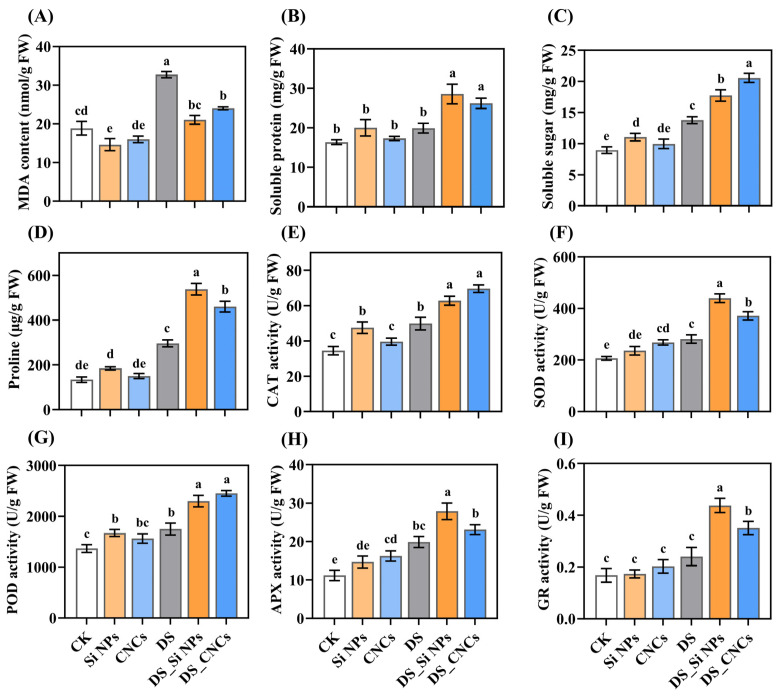
Effects of Si NPs and CNCs on malondialdehyde, osmotic regulators, and antioxidant enzyme activities in tall fescue leaves under drought stress: (**A**) malondialdehyde (MDA), (**B**) soluble protein, (**C**) soluble sugar, (**D**) proline, (**E**) catalase (CAT), (**F**) superoxide dismutase (SOD), (**G**) peroxidase (POD), (**H**) ascorbate peroxidase (APX), (**I**) glutathione reductase (GR). Data are presented as means ± standard deviation from three biological experiments. Different letters above the bars indicate significant differences among treatments based on Tukey’s HSD test (one-way ANOVA, *p* < 0.05). Treatment abbreviations: CK (control, well-watered), Si NPs (well-watered + 300 mg/L silicon nanoparticles), CNCs (well-watered + 100 mg/L cellulose nanocrystals), DS (drought stress), DS_Si NPs (drought stress + 300 mg/L silicon nanoparticles), DS_CNCs (drought stress + 100 mg/L cellulose nanocrystals).

In this study, we investigated the effects of foliar application of Si NPs and CNCs on MDA levels, osmotic adjustment substances, and antioxidant enzyme activity in tall fescue leaves under both normal and drought conditions (Figure 5). Under normal conditions, Si NPs significantly reduced MDA content by 29.01% compared to control (CK) plants (*p* < 0.05) (Figure 5A). Among osmotic adjustment substances, only Si NPs showed a significant effect on soluble sugar content, increasing it by 23.38% compared to CK plants (*p* < 0.05) (Figure 5B–D). Additionally, Si NPs significantly enhanced CAT and POD activity. In contrast, CNCs exhibited a significant effect in increasing SOD and APX activity (*p* < 0.05) (Figure 5E–H). Under drought stress, both nanomaterials effectively reduced MDA accumulation, maintained osmotic balance, and enhanced antioxidant enzyme activity. Specifically, in the drought-stressed control (DS), the highest MDA content was observed, while it was significantly reduced by 35.69% and 26.61% following Si NP and CNC treatments, respectively (*p* < 0.05) (Figure 5A). Moreover, both nanomaterials significantly increased the contents of soluble protein, soluble sugar, and proline (*p* < 0.05) (Figure 5B–D). Regarding antioxidant enzyme activity, CNCs resulted in the highest CAT and POD activities, whereas Si NPs enhanced SOD, APX, and GR activities by 56.21%, 40.29%, and 82.12%, respectively, compared to DS plants (*p* < 0.05) (Figure 5E–I).

### 2.6. Effects on Nitrogen, Phosphorus, Potassium, and Silicon Accumulation

To investigate the effects of foliar application of the two nanomaterials on nutrient element accumulation in leaves, the contents of nitrogen (N), phosphorus (P), potassium (K), and silicon (Si) were measured (Table 2). Under normal conditions, Si NP treatment significantly increased leaf K and Si contents by 21.13% and 88.98%, respectively, compared to control (CK) plants (*p* < 0.05) (Table 2). In contrast, CNC treatment significantly enhanced P content, showing a 32.28% increase relative to CK plants (*p* < 0.05) (Table 2). Under drought conditions, untreated plants (DS) exhibited the lowest nutrient element contents, especially the N content, which decreased by 32.77% compared to CK plants (Table 2). After the application of both nanomaterials, nutrient contents improved. Notably, plants treated with Si NPs reached the highest levels of N, P, K, and Si, increasing by 33.29%, 37.67%, 30.93%, and 107.08%, respectively, compared to DS plants (*p* < 0.05). CNC treatment also significantly enhanced K content (*p* < 0.05), although the improvements in other elements were less pronounced than those observed with Si NPs (Table 2).

### 2.7. Transcriptome Analysis of Leaves Under Different Treatments

To elucidate the roles of the two nanomaterials in promoting tall fescue growth and drought tolerance, transcriptome sequencing was performed on plants treated with Si NPs and CNCs for 20 days under both normal and drought conditions. After data filtering, each sample yielded at least 5.02G clean reads, with an average Q30 base quality exceeding 93.39% and GC content ranging from 53.17% to 55.07%, indicating high transcriptome data quality and consistency (Table S1). Due to the lack of a reference genome for tall fescue, de novo assembly was used to obtain unigenes.

**Figure 6:**
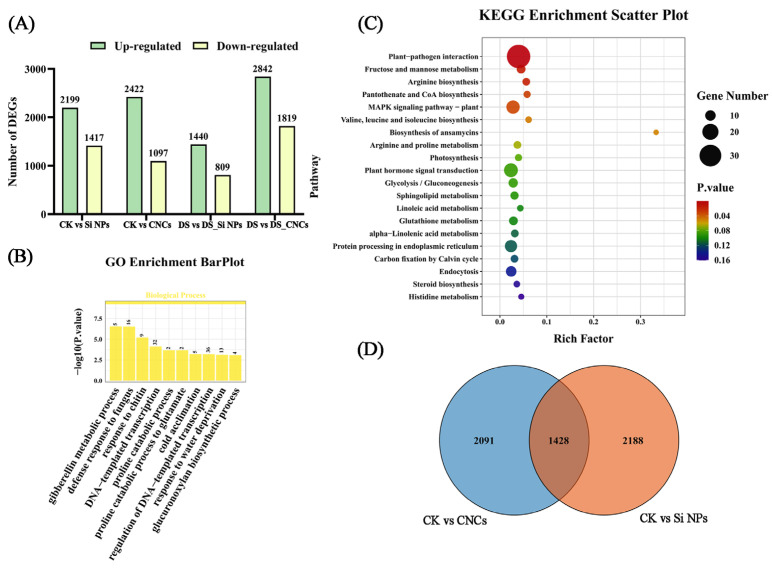
Transcriptome analysis of plants sprayed with nanomaterials and control plants under normal water conditions. (**A**) Number of DEGs, (**B**) GO enrichment analysis of 1428 DEGs shared after the application of nanomaterials, (**C**) KEGG enrichment analysis of 1428 DEGs shared after the application of nanomaterials, (**D**) Venn analysis of DEGs after the application of nanomaterials. Treatment abbreviations: CK (control, well-watered), Si NPs (well-watered + 300 mg/L silicon nanoparticles), CNCs (well-watered + 100 mg/L cellulose nanocrystals).

Under normal conditions, 1428 shared differentially expressed genes (DEGs) were identified in response to Si NP and CNC treatments (Figure 6D). Among them, 943 genes were upregulated in Si NPs, while 941 genes were upregulated in CNCs. Notably, 65.9% of the shared DEGs exhibited the same expression patterns after applying both nanomaterials. GO enrichment analysis revealed that these DEGs were primarily associated with gibberellin metabolism, defense response to fungus, and chitin response (Figure 6B). KEGG enrichment analysis indicated that they were mainly involved in pathways such as plant-pathogen interaction, photosynthesis, and plant hormone signal transduction (Figure 6C). In the photosynthesis-related pathway, genes such as chloroplast atpB gene product (*atpB*), cytochrome b-559 alpha subunit chloroplast (*Cytb559*), and photosystem II 10 kDa polypeptide (*PSBR*) were upregulated under both nanomaterial treatments. *AtpB* and *PSBR* exhibited more significant upregulation under Si NP treatment, reaching 1.61-fold and 4.89-fold levels compared to CK plants. At the same time, *Cytb559* showed greater upregulation under CNC treatment (Table S2). Additionally, in the plant hormone signal transduction pathway, the key gene *SAUR32-like* was upregulated by 45.11-fold in CNCs and 21.39-fold in Si NPs (Table S2).

**Figure 7:**
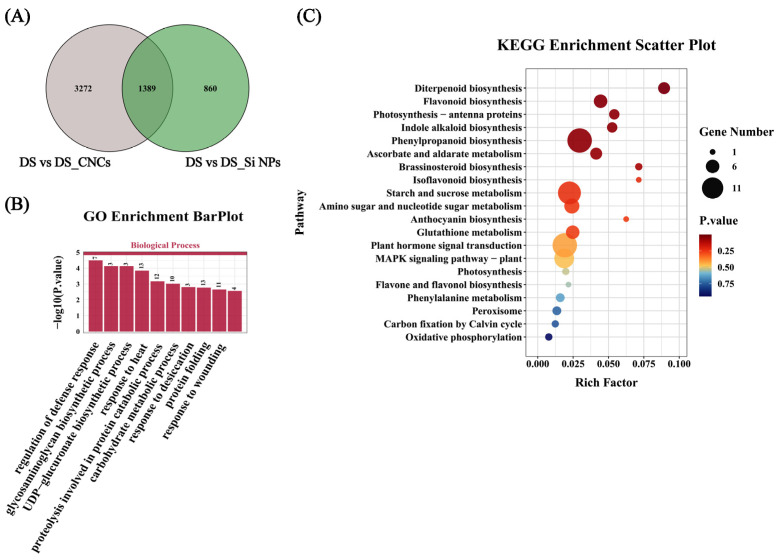
Transcriptome analysis of plants sprayed with nanomaterials and control plants under drought stress. (**A**) Venn analysis of DEGs after the application of nanomaterials under drought stress, (**B**) GO enrichment analysis of 1389 shared DEGs under drought stress after the application of nanomaterials, (**C**) KEGG enrichment analysis of 1389 shared DEGs under drought stress after the application of nanomaterials. Treatment abbreviations: DS (drought stress), DS_Si NPs (drought stress + 300 mg/L silicon nanoparticles), DS_CNCs (drought stress + 100 mg/L cellulose nanocrystals).

Under drought stress, treatments with Si NPs and CNCs revealed 1389 shared DEGs with consistent expression patterns (Figure 7A). GO enrichment analysis revealed that these DEGs were mainly involved in biological processes such as heat response, defense response regulation, and response to desiccation (Figure 7B). KEGG enrichment analysis showed that the DEGs were primarily involved in metabolic pathways including diterpenoid biosynthesis, flavonoid biosynthesis, and photosynthesis—antenna proteins (Figure 7C). This suggests that both nanomaterials may improve drought tolerance in tall fescue through a common mechanism. Genes related to photosynthesis antenna proteins, such as chlorophyll a-b binding protein of LHCII type 1 (*CAB-M9*) and light-harvesting chlorophyll a/b-binding protein Lhcb1 (*CAB1*), were included in the 888 upregulated DEGs (Table S2). Additionally, key genes involved in ROS scavenging, such as *APX1*, *GST3*, *CAT*2, and *SOD1*, were upregulated following treatment with both nanomaterials. Specifically, *APX1*, *GST3*, and *SOD1* were upregulated to a greater extent in Si NP-treated plants, with increases of 1.41, 27.51, and 1.91 times, respectively, compared to DS plants. In contrast, *CAT2* showed a greater increase in CNC-treated plants, with a 22.51-fold increase compared to DS plants (Table S2).

Moreover, under drought stress, the Si NP-treated plants shared 2249 DEGs with untreated plants, 860 of which were specific to Si NPs (excluding shared DEGs), while CNCs resulted in 3272 specific DEGs. KEGG enrichment analysis revealed that Si NP-specific DEGs were mainly involved in pathways such as diterpenoid biosynthesis, MAPK signaling pathway, and plant hormone signal transduction (Figure S2). The LRR receptor-like serine/threonine-protein kinase (*ERECTA*), a key gene in the MAPK signaling pathway, showed a 4.02-fold increase in expression compared to DS plants. In contrast, pathogenesis-related protein (*PRP*), involved in the plant-pathogen interaction pathway, showed a 68.06-fold increase compared to DS plants (Table S2). KEGG enrichment analysis further revealed that the specific DEGs in CNC-treated plants under drought stress were mainly involved in pathways such as phenylpropanoid biosynthesis, linoleic acid metabolism, and isoflavonoid biosynthesis (Figure S3), with 3191 of these genes (84% of total specific genes) upregulated under drought stress. Notably, CNC treatment upregulated genes related to plant cell wall synthesis and metabolism, such as cinnamoyl CoA reductase (*CCR*) and beta-glucosidase 5-like (*BGLU5-like*), with expression levels increasing by 3.87-fold and 18.22-fold, respectively, compared to DS plants (Table S2).

To further validate the reliability of the transcriptome data, we randomly selected 8 DEGs for qRT-PCR analysis. The qPCR expression profiles of all randomly selected genes were consistent with the RNA-seq data, indicating that the RNA sequencing data are highly reliable and accurate (Figure S4).

## 3. Discussion

Due to the unique growing conditions of steep slopes, water scarcity presents a significant challenge to the growth and development of tall fescue, affecting ecological restoration outcomes. In the context of climate change, exploring efficient methods to enhance the drought tolerance of slope-protecting plants is of utmost importance. This study found that drought significantly inhibited the growth of tall fescue, reducing biomass and increasing oxidative and osmotic stress. The results showed that exogenous application of Si NPs and CNCs promoted drought tolerance and increased biomass by enhancing leaf photosynthetic performance, osmotic regulation, antioxidant enzyme activity, and nutrient absorption. Notably, Si NPs were more effective in achieving these results.

Drought-induced symptoms are most apparent at the morphological level, hindering normal plant growth [21]. In this study, Si NPs significantly improved the growth parameters of tall fescue under drought conditions, including leaf length, root length, and biomass (Figure 2). Similar results have been confirmed in other studies [22,23]. Under drought, plant roots are the first to perceive insufficient soil moisture and make adaptive adjustments by absorbing water and nutrients from deeper soil layers, thus enhancing drought tolerance [24]. Studies have shown that overexpression of the *PRP* gene can promote root elongation in poplar [25]. In our study, Si NPs resulted in a 68.06-fold increase in *PRP* gene expression compared to the DS plants (Table S2), leading to a 19.69% increase in root length (Figure 2). In contrast, CNC treatment also had a certain promoting effect on growth. However, the difference compared to DS plants was insignificant (Figure 2). CNCs mainly alleviate drought stress by improving the strength and stability of the plant cell wall. The *BGLU5-like* gene is involved in the lignification and secondary metabolic processes of the cell wall [26], while the *CCR* gene, which encodes cinnamoyl-CoA reductase, is a key enzyme in lignin biosynthesis and can promote lignin synthesis [27,28]. In this study, CNC treatment led to an 18.22-fold and 3.87-fold increase in the expression of *BGLU5-like* and *CCR*, respectively, compared to DS plants (Table S2), and this effect was more pronounced than with Si NPs. This suggests that CNCs increase lignin synthesis, enhance cell wall stability, reduce water loss, and thus protect plant cells from drought-induced damage.

Drought stress suppresses plant photosynthesis by reducing the content of photosynthetic pigments, leading to decreased photosynthetic products and inhibiting plant growth and development [29]. In this study, Si NPs significantly increased the contents of chlorophyll a and b (Figure 3), which may be due to the Si-mediated increase in antioxidant enzyme activity that maintains reactive oxygen species (ROS) homeostasis, thereby reducing the loss of photosynthetic pigments [30]. Additionally, studies have shown that drought stress significantly reduced gas exchange parameters (including photosynthetic rate, stomatal conductance, and transpiration rate) in broad beans, and applying Si NPs effectively improved this phenomenon [31]. Our results were consistent with these findings (Figure 3). Although direct evidence of the CNC effect on photosynthesis is lacking, it likely shares a mechanism similar to Si NPs. In this study, photosynthesis-related genes, such as *CAB-M9* and *CAB1*, were upregulated in Si NP and CNC treatments (Table S2). These genes are involved in light energy capture and transfer, photoprotection, and thylakoid membrane structure maintenance, all crucial for photosynthesis [32,33]. Furthermore, Zou et al. [34] found that plants overexpressing the *PIP2-7* gene showed a slower rate of chlorophyll content reduction under drought stress. In our study, Si NP treatment upregulated *PIP2-7* gene expression by 13.93-fold. In contrast, CNC treatment increased it by 12.28-fold (Table S2). Under normal conditions, we also observed that both nanomaterials upregulated the expression of important functional genes related to photosynthesis, such as *atpB*, *Cytb559*, and *PSBR* [35,36,37], which may be closely related to their growth-promoting characteristics (Table S2).

Long-term drought not only reduces leaf photosynthetic pigment levels but also alters the apparent morphology and microscopic structure of leaves. It is generally believed that thicker leaves have better water storage capacity and drought tolerance [38]. This study found that Si NP treatment significantly increased leaf thickness and related structures (Table 1). Avestan et al. [39] showed that Si NPs reduce the negative effects of salt stress by increasing leaf thickness and providing higher water content. The increase in leaf thickness induced by Si NPs is partly due to the deposition of silicon beneath the cuticle and cell wall, forming a silica-cellulose bilayer and silica-cuticle bilayer on the leaf surface [40]. On the other hand, Si NP treatment under drought conditions increased leaf water content and water potential [41]. Additionally, the increase in vascular bundle area is closely related to drought tolerance. Under drought stress, plants typically enlarge the vascular bundle area to improve water transport efficiency [42]. Our study found that Si NPs further increased the vascular bundle area (Figure 4). In contrast, we hypothesize that the mechanism of CNCs may differ from that of Si NPs. Its high mechanical strength enhances cell wall stability, preventing cell deformation due to water shortage. In contrast, its water absorption and retention properties may form a “water film” on the leaf surface, reducing water evaporation. Therefore, CNCs may mainly protect leaf structure and maintain cell integrity and function by enhancing the mechanical stability of the cell wall and changing its physical properties without causing significant changes in leaf structure. However, the exact interaction mechanism of CNCs with the leaf still requires further investigation.

Plants accumulate osmotic regulatory substances such as proline, soluble sugars, and soluble proteins to cope with drought stress. Importantly, proline not only directly enhances drought tolerance by maintaining cell turgor and osmotic balance but also functions as a reactive oxygen species (ROS) scavenger, thereby reducing oxidative damage. Furthermore, it stabilizes protein structures and forms a desiccation protective layer, which helps to alleviate cell collapse induced by drought. Additionally, the accumulation of proline may positively regulate the expression of stress-responsive genes, such as *P5CS* and *SOD*, thereby establishing a feedback loop that enhances stress tolerance [43,44]. In this study, both Si NPs and CNCs significantly increased the content of proline, soluble proteins, and soluble sugars under drought stress (Figure 5). Si NPs have been shown to improve plant osmotic regulation capacity [45,46]. Although there is a lack of relevant studies on CNCs, they may enhance drought tolerance through a similar mechanism. Drought leads to the excessive production of ROS, exacerbating lipid peroxidation and damaging membrane integrity [47]. MDA is a marker of lipid peroxidation [48], and in this study, Si NPs reduced MDA content by 35.69%, consistent with the findings of Wahi et al. [49]. CNCs also showed a similar effect (Figure 5). Plants activate antioxidant defense mechanisms to detoxify ROS and maintain cellular stability [50]. In this study, Si NPs reduced oxidative damage by increasing the activity of SOD, GR, CAT, and POD (Figure 5), which is consistent with the findings of Liu et al. [51], while CNCs also exhibited a similar effect. APX catalyzes the conversion of ascorbic acid (AsA) to dehydroascorbic acid and participates in the AsA-glutathione cycle, where dehydroascorbate reductase (DHAR) eliminates H_2_O_2_ [52]. In this study, both nanomaterials increased APX activity under drought stress and upregulated the expression of *DHAR2*, *APX1*, and *GST3* genes (Figure 5, Table S2). Additionally, we noted that the expression of *SOD1* was more upregulated in Si NP-treated plants, while *CAT2* expression was significantly higher under CNC treatment, consistent with the physiological results (Table S2). These results suggest that Si NPs and CNCs significantly enhance the plant’s tolerance to oxidative stress by upregulating the antioxidant defense system and relevant gene expression, alleviating drought stress damage, and promoting plant growth.

Drought inhibits the absorption and transport of nutrients by plant roots, severely affecting plant growth and development [53]. In this study, Si NP treatment significantly increased the nitrogen content in the leaves, showing a 33.29% increase compared to the DS plants, with improvements also observed in phosphorus and potassium content (Table 2). This result aligns with the study by Alsaeedi et al. [54], who found that Si NP application increased potassium content in both the leaves and roots of cucumber (*Cucumis sativus*). Higher potassium levels can stimulate root development [55,56], which may be another reason why Si NPs significantly increased root length. Additionally, Si NPs significantly increased the silicon concentration in the leaves (Table 2), consistent with previous studies [45,57]. Transcriptome analysis showed that Si NP treatment upregulated the expression of the *Lsi2* gene, which plays an important role in the long-distance transport of silicon [58,59]. Therefore, exogenous Si NPs not only increase silicon concentrations in the leaves but may also enhance silicon accumulation by promoting long-distance transport through upregulating *Lsi2* gene expression. In contrast, CNC treatment only significantly increased potassium content in the leaves (Table 2), with minimal effects on nitrogen, phosphorus, and silicon content. This may be because CNCs primarily promote potassium absorption by enhancing cell membrane stability and adsorption capacity, which differs from the absorption mechanisms of nitrogen, phosphorus, and other elements and is more restricted under drought stress. Therefore, future studies should further explore the specific mechanisms of nutrient element absorption and utilization mediated by CNCs under stress conditions to reveal its potential regulatory pathways.

## 4. Materials and Methods

### 4.1. Experimental Materials

Tall fescue (*Festuca arundinacea* Schreb.) seeds were purchased from Tai’an Boqi Seed Industry Co., Ltd. (Tai’an, China), with purity > 98% and moisture content < 13%.

Silicon nanoparticles (Si NPs) and cellulose nanocrystals (CNCs) were purchased from Jiangsu Xianfeng Nanomaterials Technology Co., Ltd. (Nanjing, China). According to the technical documentation provided by the supplier, Si NPs were synthesized via a sol–gel method (purity > 98%) using tetraethyl orthosilicate (TEOS) as the silicon precursor. The hydrolysis of TEOS was conducted in an ethanol solution at pH 3.5 and 25 °C for 2 h. Subsequently, the pH was adjusted to 8.0 using ammonia solution, and the temperature was elevated to 50 °C to facilitate polycondensation for 4 h, yielding silica gel. The gel was aged for 48 h, vacuum-dried at 60 °C, and calcined at 500 °C for 3 h. Finally, high-purity silica nanoparticles were obtained through ball milling and sieving. In contrast, CNCs were prepared via acid hydrolysis. Cotton linters were treated with 60–65% sulfuric acid at 45–55 °C for 30–120 min to selectively remove amorphous cellulose domains. The hydrolyzed product was purified by centrifugation and dialysis, followed by ultrasonication to disperse the material into a nanocrystalline suspension. The CNCs were ultimately collected through freeze-drying. Both nanomaterials were sputtered and gold-coated for 30 s and then examined using a JEM1200EX transmission electron microscope (TEM, JEOL, Akishima, Japan). The particle size of Si NPs and the length of CNCs were measured using ImageJ (version 1.54). The zeta potentials of the two nanomaterials were measured using a Zetasizer Nano ZS90 (Malvern Panalytical, Malvern, UK). Samples were dispersed in deionized water, ultrasonicated, and analyzed at 25 °C with three replicates. The functional groups of the materials were analyzed using a Nicolet IS10 Fourier-transform infrared spectrometer (FTIR, Nicolet, Madison, WI, USA), with a scanning range of 500–4000 cm^−1^. X-ray diffraction (XRD) analysis was performed using a Rigaku Miniflex600 diffractometer (Rigaku, Akishima Japan), scanning from 5° to 80° (2θ) at a scan rate of 2°/min.

### 4.2. Experimental Design

The experiment was conducted in a greenhouse at the College of Soil and Water Conservation Science and Engineering, Northwest A&F University, Yangling, China (34°27′ N, 108°07′ E), from July to September 2024. The temperature was maintained at 25 ± 5 °C and humidity at 70 ± 5%, with a light cycle of 16 h of light and 8 h of darkness. Uniform and plump tall fescue seeds were selected, with 50 seeds sown per pot (substrate: a mixture of peat, vermiculite, and perlite in a 1:1:1 ratio). After germination, 30 healthy seedlings per pot were kept for further cultivation. When the seedlings reached 15–20 cm, a 10-day concentration screening experiment was performed. The plants were divided into six treatments with the following experimental conditions: (i) Normal control (CK): Plants were sprayed with double-distilled water (DDW) and maintained at normal soil moisture (65–75% field capacity); (ii) Si NP treatments: Plants were sprayed with Si NP solutions at concentrations of 50, 100, 200, 300, and 500 mg/L under normal soil moisture conditions; (iii) CNC treatments: Plants were sprayed with CNC solutions at concentrations of 25, 50, 100, 200, and 300 mg/L under normal soil moisture conditions; (iv) Drought stress control (DS): Plants were grown under drought conditions (35–45% field capacity); (v) DS_Si NP treatments: Plants were sprayed with Si NP solutions at the same concentrations as treatment ii and grown under drought conditions; (vi) DS_CNC treatments: Plants were sprayed with CNC solutions at the same concentrations as treatment iii and grown under drought conditions. Each treatment was repeated three times. Before spraying, the nanomaterials were diluted with DDW and sonicated for 30 min. After adding 0.05% Tween-20, the solution was evenly sprayed on the leaves, with applications every three days. Soil moisture was replenished daily using the gravimetric method, and plastic film was used to cover the soil surface to prevent solution leakage. After 10 days, plant growth and physiological parameters were measured.

The concentration screening results (Figures S5 and S6) indicated that the growth-promoting effects of Si NPs and CNCs on tall fescue exhibited distinct concentration-dependent patterns. For the Si NP treatment group (50–500 mg/L), the peak promoting effect occurred at 300 mg/L under both normal and drought conditions. In contrast, the CNC treatment group (25–300 mg/L) showed a typical hormesis effect, where maximum enhancement of growth and physiological parameters was achieved at 100 mg/L, while higher concentrations resulted in significant inhibitory effects. Based on these concentration-response trends, this study selected 300 mg/L Si NPs and 100 mg/L CNCs as the optimal treatment concentrations. After an additional 10-day treatment (for a total of 20 days), samples were collected. Functional leaves of medium size were selected, and samples were divided into three parts: one portion was stored at −20 °C for physiological and biochemical measurements, another part was flash-frozen in liquid nitrogen and stored at −80 °C for transcriptomic analysis, and the remaining portion was dried and ground for analysis of nitrogen, phosphorus, potassium, and silicon content. Additionally, root samples were collected to measure root length.

### 4.3. Growth Parameters and Biomass Measurement

The height of the aerial parts, leaf length, and root length were measured using a millimeter ruler. Leaf width and stem diameter were measured using a caliper. Five plants were grouped for biomass measurement, and the whole plant was harvested. The leaves and roots were washed with distilled water to remove surface impurities and soil, then blanched in an oven at 105 °C for 15 min. The samples were then dried to a constant weight at 65 °C in a constant-temperature drying oven and weighed to determine biomass.

### 4.4. Chlorophyll and Gas Exchange Parameter Measurement

Medium-sized functional leaves were selected, and chlorophyll pigments were extracted with 95% ethanol (*v*/*v*) at 4 °C. Absorbance was measured at 665 nm, 649 nm, and 470 nm to calculate chlorophyll a, chlorophyll b, and carotenoid contents [60]. Leaf gas exchange parameters were measured following Zhang et al. [61] using an Li-6800 portable photosynthesis system (LI-COR, Lincoln, NE, USA). Measurements were taken on sunny days between 9:00 and 11:00 AM, recording photosynthetic rate, transpiration rate, and stomatal conductance.

### 4.5. Leaf Anatomical Structure Analysis

Leaf anatomical structure was observed using the paraffin section method. Medium-sized functional leaves were selected, and 1–1.5 mm thick leaf sections containing the midrib were cut and fixed in 50% formalin-acetic acid-alcohol (FAA) solution for 24 h [62]. The samples were embedded in paraffin after a series of ethanol gradient dehydrations. Thin sections (8 μm) were cut using a Leica RM2235 microtome (Leica, Wetzlar, Germany). The sections were stained with toluidine blue for 2–5 min, washed with distilled water, and then immersed in xylene for 10 min. The sections were scanned using a Pannoramic desk P1000 slide scanner (3DHISTECH Ltd., Budapest, Hungary). Leaf anatomical structure parameters, including leaf thickness, upper and lower epidermal thickness, upper and lower epidermis cuticle thickness, and vascular bundle area, were observed and analyzed using CaseViewer (3DHISTECH, Budapest, Hungary, version 2.3).

### 4.6. MDA, Osmotic Regulators, and Antioxidant Enzyme Activity Measurement

MDA content was measured using the thiobarbituric acid (TBA) method [63]. Soluble sugar content was determined using the anthrone colorimetric method [64]. Soluble protein content was measured using the Coomassie Brilliant Blue G-250 staining method [65]. Proline content was determined using the acidic ninhydrin [66]. Antioxidant enzyme activities were measured using commercial assay kits (Solarbio, Beijing Solarbio Science & Technology Co., Ltd., Beijing, China, Product No. BC0170, BC0090, BC0200, BC1160, BC0220). The activities of superoxide dismutase (SOD, EC1.15.1.1), peroxidase (POD, EC1.11.1.7), catalase (CAT, EC1.11.1.6), glutathione reductase (GR, EC1.8.1.7), and ascorbate peroxidase (APX, EC1.11.1.11) were measured by spectrophotometry. A total of 0.1 g of sample was collected for each measurement. Enzyme activity for each sample was determined according to the kit instructions, and each sample was tested in triplicate.

### 4.7. Determination of Nitrogen, Phosphorus, Potassium, and Silicon Content

After drying the leaf samples to a constant weight, they were ground and passed through a 0.25 mm sieve. To obtain the test solution, the plant nitrogen, phosphorus, and potassium contents were determined using the H_2_SO_4_-H_2_O_2_ digestion method. Nitrogen content was measured by the Kjeldahl method, phosphorus content by the molybdenum-antimony anti-spectrophotometric method, and potassium content by atomic absorption spectrometry [67]. Silicon content was determined using the molybdenum blue colorimetric method [57].

### 4.8. RNA Extraction, Library Construction, and Sequencing

Leaf samples from six treatments were collected, namely CK, Si NPs, CNCs, DS, DS_Si NPs, and DS_CNCs. After thorough grinding in liquid nitrogen, total RNA was extracted using the Plant RNA Kit (R6827, Omega, Guangzhou, China). The concentration and purity of the RNA were measured using a NanoDrop ND-1000 spectrophotometer (Thermo Fisher Scientific, Waltham, MA, USA), and RNA integrity was assessed using an Agilent 2100 BioAnalyzer (Agilent Technologies, Santa Clara, CA, USA). Qualified samples were transported on dry ice to LC-BIO Technology Co., Ltd. in Hangzhou, China, for library construction and sequencing.

For library construction, mRNA with polyA tails was specifically captured using oligo(dT) magnetic beads (Dynabeads Oligo (dT), cat. 25-61005, Thermo Fisher, Waltham, MA, USA) through two rounds of purification. The captured mRNA was fragmented at high temperatures using a magnesium RNA fragmentation module (NEBNext^®^ Magnesium RNA Fragmentation Module, cat. E6150S, Ipswich, MA, USA) for 5–7 min at 94 °C. The fragmented RNA was reverse transcribed into cDNA using Invitrogen SuperScript™ II Reverse Transcriptase (cat. 1896649, Carlsbad, CA, USA). Subsequently, double-strand synthesis was performed using *E. coli* DNA polymerase I (NEB, cat. M0209, Ipswich, MA, USA) and RNase H (NEB, cat. M0297, Ipswich, MA, USA) to convert the RNA-DNA hybrid duplex into a double-stranded DNA while incorporating dUTP Solution (Thermo Fisher, cat. R0133, Carlsbad, CA, USA) into the second strand. The ends of the double-stranded DNA were blunted, and an A base was added to each end to facilitate ligation with adaptors containing T overhangs. Magnetic bead-based size selection and purification were then conducted. The second strand was digested using the UDG enzyme (NEB, cat. M0280, Ipswich, MA, USA). PCR amplification was performed under the following conditions: initial denaturation at 95 °C for 3 min, followed by eight cycles of 98 °C for 15 s, 60 °C for 15 s, and 72 °C for 30 s, with a final extension at 72 °C for 5 min. This process generated a strand-specific library with fragment sizes of 300 bp ± 50 bp. Finally, the library was sequenced using the Illumina Novaseq™ 6000 platform (LC-BIO Technology Co., Ltd., Hangzhou, China) in a paired-end mode (PE150) according to the standard operating procedures.

### 4.9. Transcriptomic Data Analysis

Initially, reads with adapter contamination, low quality, and undetermined bases were removed using Cutadapt (version 1.9). Subsequently, FastQC (version 0.10.1) was employed to validate sequence quality, including the Q20, Q30, and GC content of the clean data. All downstream analyses were based on high-quality, clean data. The transcriptome was de novo assembled using Trinity (version 2.15). Trinity clusters transcripts based on shared sequence content. These transcript clusters were loosely termed “genes”. The longest transcript within each cluster was selected as the “gene” sequence (Unigene). All assembled Unigenes were aligned against the non-redundant (Nr) protein database, Gene Ontology (GO), SwissProt, Kyoto Encyclopedia of Genes and Genomes (KEGG), and eggNOG databases using DIAMOND (version 2.0.15) with a threshold of E < 0.00001. The expression levels of Unigenes were analyzed using Salmon (version 1.9.0) by calculating transcripts per kilobase of exon model per million mapped reads (TPM). Differentially expressed Unigenes were identified using the R package edgeR (version 3.40.2), with criteria of log_2_(fold change) > 2 or log_2_(fold change) < −2 and false discovery rate (FDR) < 0.05.

### 4.10. Quantitative Real-Time PCR Validation

Gene expression changes were measured using the quantitative real-time polymerase chain reaction (qRT-PCR) method to validate the transcriptomic results, with eight differentially expressed genes (DEGs) selected from each sample. The primer sequences for these genes are listed in Table S3. Total RNA was isolated from leaf tissues of three biological replicates using the Plant RNA Kit (R6827, Omega, Guangzhou, China). First-strand cDNA synthesis was performed using the EasyScript^®^ One-Step gDNA Removal and cDNA Synthesis SuperMix (AE311, TransGen, Beijing, China) according to the manufacturer’s instructions. qRT-PCR was conducted using the Qantstudio5 Real-Time PCR System with 2×SYBR green qPCR Mix (PC3302, Aidlab, Beijing, China) as the fluorescent dye. The qRT-PCR results were calculated using the 2^−ΔΔCt^ method [68], with the actin gene as the internal control.

### 4.11. Statistical Analysis

For data analysis, SPSS version 25.0 (SPSS, Inc., Chicago, IL, USA) statistical software was used, and one-way analysis of variance (ANOVA) was performed to analyze the significance of differences between different treatments at a probability level of 5%. Multiple comparisons of means were carried out using Tukey’s HSD post hoc test. Graphs were created using R version 4.0.4 and GraphPad Prism version 10.0 (GraphPad Software, San Diego, CA, USA).

## 5. Conclusions

Foliar application of silicon nanoparticles and cellulose nanocrystals promoted the growth and biomass accumulation of tall fescue under drought stress, with silicon nanoparticles showing better results than cellulose nanocrystals. Both nanomaterials improved drought tolerance by enhancing photosynthesis, osmotic regulation, and antioxidant enzyme activity and upregulating the expression of related functional genes, demonstrating similar mechanisms of action. Furthermore, silicon nanoparticles enhanced drought tolerance by stimulating root growth, improving nutrient absorption, and improving leaf structure. At the same time, cellulose nanocrystals relied on their water retention capacity and regulation of cell wall-related genes to maintain cellular stability and protect against drought damage. However, future studies should further explore their long-term ecological effects and mechanisms in enhancing plant tolerance, especially cellulose nanocrystals, which, to our knowledge, is the first report on using cellulose nanocrystals in foliar application to alleviate plant drought stress.

## Figures and Tables

**Table 1:** Statistical analysis of leaf anatomical structure parameters.

Treatments	Leaf Thickness (μm)	Ue Thickness (μm)	Le Thickness (μm)	Uec Thickness (μm)	Lec Thickness (μm)	VB Area (μm^2^)
CK	335.04 ± 11.3 cd	14.28 ± 1.54 d	23.81 ± 2.39 c	1.43 ± 0.21 c	2.18 ± 0.38 c	3892.21 ± 919.86 c
Si NPs	462.65 ± 11.16 a	18.34 ± 4 bcd	37.14 ± 3.81 abc	2.68 ± 0.54 ab	4.05 ± 0.45 ab	6126.82 ± 783.16 abc
CNCs	382.37 ± 27.09 bc	15.12 ± 3.01 cd	35.98 ± 8.92 abc	1.85 ± 0.18 bc	3.22 ± 0.58 bc	7700.13 ± 1330.01 ab
DS	263.32 ± 17.61 e	24.82 ± 4.64 abc	32.23 ± 5.74 bc	2.18 ± 0.27 bc	2.73 ± 0.43 bc	4779.6 ± 692.31 c
DS_Si NPs	418.7 ± 30.39 ab	30.31 ± 2.89 a	52 ± 7.59 a	3.28 ± 0.63 a	5.1 ± 0.76 a	8200.35 ± 917.88 a
DS_CNCs	289.12 ± 23.56 de	26.81 ± 4.63 ab	42.45 ± 4.08 ab	2.34 ± 0.34 abc	4.04 ± 0.44 ab	5496.32 ± 805.68 bc

Note: Ue, upper epidermal; Le, lower epidermal; Uec, upper epidermal cuticle; Lec, lower epidermal cuticle; VB, vascular bundle. Data are presented as means ± standard deviation from three biological experiments. Different letters within the table indicate significant differences among treatments based on Tukey’s HSD test (one-way ANOVA, *p* < 0.05). Treatment abbreviations: CK (control, well-watered), Si NPs (well-watered + 300 mg/L silicon nanoparticles), CNCs (well-watered + 100 mg/L cellulose nanocrystals), DS (drought stress), DS_Si NPs (drought stress + 300 mg/L silicon nanoparticles), DS_CNCs (drought stress + 100 mg/L cellulose nanocrystals).

**Table 2:** Effects of Si NPs and CNCs on N, P, K, and Si content in tall fescue leaves under drought stress.

Treatments	N Concentration (g/kg DW)	P Concentration (g/kg DW)	K Concentration (g/kg DW)	Si Concentration (g/kg DW)
CK	20.48 ± 1.31 ab	4.81 ± 0.36 bc	5.01 ± 0.42 b	3.83 ± 0.26 bc
Si NPs	23.22 ± 1.35 a	5.45 ± 0.56 ab	6.07 ± 0.26 a	7.24 ± 0.27 a
CNCs	21.42 ± 0.73 a	6.39 ± 0.53 a	5.17 ± 0.35 b	4.67 ± 0.46 b
DS	13.77 ± 0.93 d	3.44 ± 0.13 d	3.99 ± 0.2 c	3.25 ± 0.12 c
DS_Si NPs	18.35 ± 1.05 bc	4.73 ± 0.25 bc	5.22 ± 0.16 b	6.72 ± 0.49 a
DS_CNCs	15.98 ± 0.29 cd	4.25 ± 0.39 cd	4.86 ± 0.29 b	3.53 ± 0.39 c

Note: Nitrogen (N), phosphorus (P), potassium (K), silicon (Si). Data are presented as means ± standard deviation from three biological experiments. Different letters within the table indicate significant differences among treatments based on Tukey’s HSD test (one-way ANOVA, *p* < 0.05). Treatment abbreviations: CK (control, well-watered), Si NPs (well-watered + 300 mg/L silicon nanoparticles), CNCs (well-watered + 100 mg/L cellulose nanocrystals), DS (drought stress), DS_Si NPs (drought stress + 300 mg/L silicon nanoparticles), DS_CNCs (drought stress + 100 mg/L cellulose nanocrystals).

## Data Availability

The raw data generated in this study have been deposited in the Gene Expression Omnibus (GEO: GSE296395) database at the National Center for Biotechnology Information (NCBI) and are publicly accessible via https://www.ncbi.nlm.nih.gov/geo/query/acc.cgi?acc=GSE296395 (accessed: 8 May 2025).

## Supplementary Materials

The following supporting information can be downloaded at https://www.mdpi.com/article/10.3390/plants14101461/s1: Figure S1: Analysis of size distribution and zeta potential; Figure S2: GO and KEGG enrichment analysis of unique genes in tall fescue plants treated and untreated with silicon nanoparticles (Si NPs) under normal and drought stress conditions; Figure S3: GO and KEGG enrichment analysis of unique genes in tall fescue plants treated and untreated with cellulose nanocrystals (CNCs) under normal and drought stress conditions; Figure S4: Validation results using qRT-PCR; Figure S5: Effect of spraying different concentrations of silicon nanoparticles (Si NPs) and cellulose nanocrystals (CNCs) on the growth height, stem diameter, leaf length, and leaf width of tall fescue under normal (A) and drought (B) conditions; Figure S6: Effect of spraying different concentrations of silicon nanoparticles (Si NPs) and cellulose nanocrystals (CNCs) on the malondialdehyde (MDA) content and the activities of catalase (CAT), superoxide dismutase (SOD), and peroxidase (POD) in tall fescue under normal (A) and drought (B) conditions; Table S1: Sequencing statistics for this study; Table S2: List of upregulated genes associated with nanomaterial addition; Table S3: Primers used in qRT-PCR.

## Abbreviations

The following abbreviations are used in this manuscript:

Si NPs	Silicon nanoparticles
CNCs	Cellulose nanocrystals
TEM	Transmission electron microscope
FTIR	Fourier-transform infrared spectrometer
MDA	Malondialdehyde
CAT	Catalase
SOD	Superoxide dismutase
POD	Peroxidase
APX	Ascorbate peroxidase
GR	Glutathione reductase
N	Nitrogen
P	Phosphorus
K	Potassium
Si	Silicon
ROS	Reactive oxygen species
DHAR	Dehydroascorbate reductase
DDW	Double distilled water
FAA	Formalin-acetic acid-alcohol
TBA	Thiobarbituric acid
qRT-PCR	Quantitative real-time polymerase chain reaction
XRD	X-ray diffraction
