# Supplementary material for: Comparative Study on the Effects of Silicon Nanoparticles and Cellulose Nanocrystals on Drought Tolerance in Tall Fescue (Festuca arundinacea Schreb.)
Source: Plants (Basel). 2025 May 14;14(10):1461. doi: 10.3390/plants14101461 (PMC12114911; doi:10.3390/plants14101461)
Supplement: Supplementary file 1 [file plants-14-01461-s001.zip › plants-3617337-supplementary.pdf]

Comparative Study on the Effects of Silicon Nanoparticles and Cellulose Nanocrystals on Drought Tolerance in Tall Fescue (*Festuca arundinacea* Schreb.)

Meng Li [1], Sile Hu [1], Xulong Bai [1], Jie Ren [1], Kanliang Tian [1,*], Huili Zhang [2,*], Zhilong Zhang [3], Vanquy Nguyen [4]

[1] College of Soil and Water Conservation Science and Engineering, Northwest A&F University, Yangling 712100, China; limeng971207@163.com (M. L.); husilenwsuaf@163.com (S. H.); bxl_nwafu@163.com (X. B.); 13150103148@163.com (J. R.)
[2] College of Water Resources and Architectural Engineering, Northwest A&F University, Yangling 712100, China;
[3] College of Forestry, Northwest A&F University, Yangling, 712100, China; zhangzl@nwafu.edu.cn (Z. Z.)
[4] Southern Branch of Joint Vietnam-Russia Tropical Science and Technology Research Center, Hochiminh, 740500, Vietnam. quynguyenvan45@gmail.com (V. N.)

* Correspondence: tiankanliang@163.com, (K. T.); huilizhang163@163.com, (H. Z.)

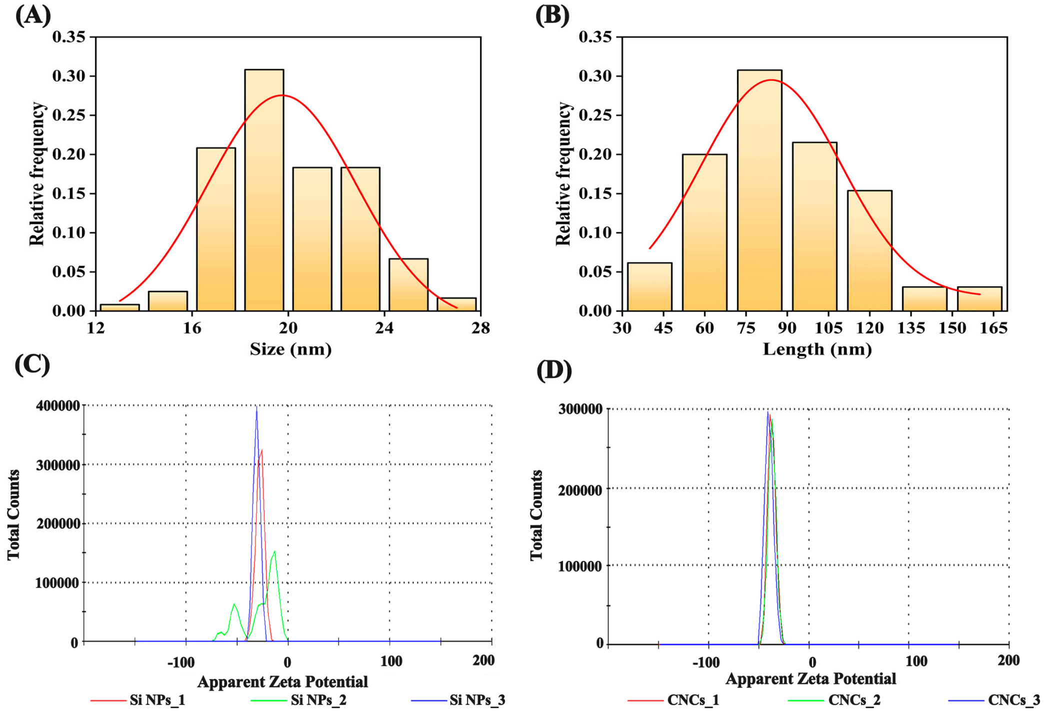

Figure S1. Analysis of size distribution and zeta potential: (A) zeta potential of silicon nanoparticles (Si NPs), (B) zeta potential of cellulose nanocrystals (CNCs), (C) particle size distribution of Si NPs, (D) length distribution of CNCs (Data are presented as mean ± standard deviation, *n*=3).

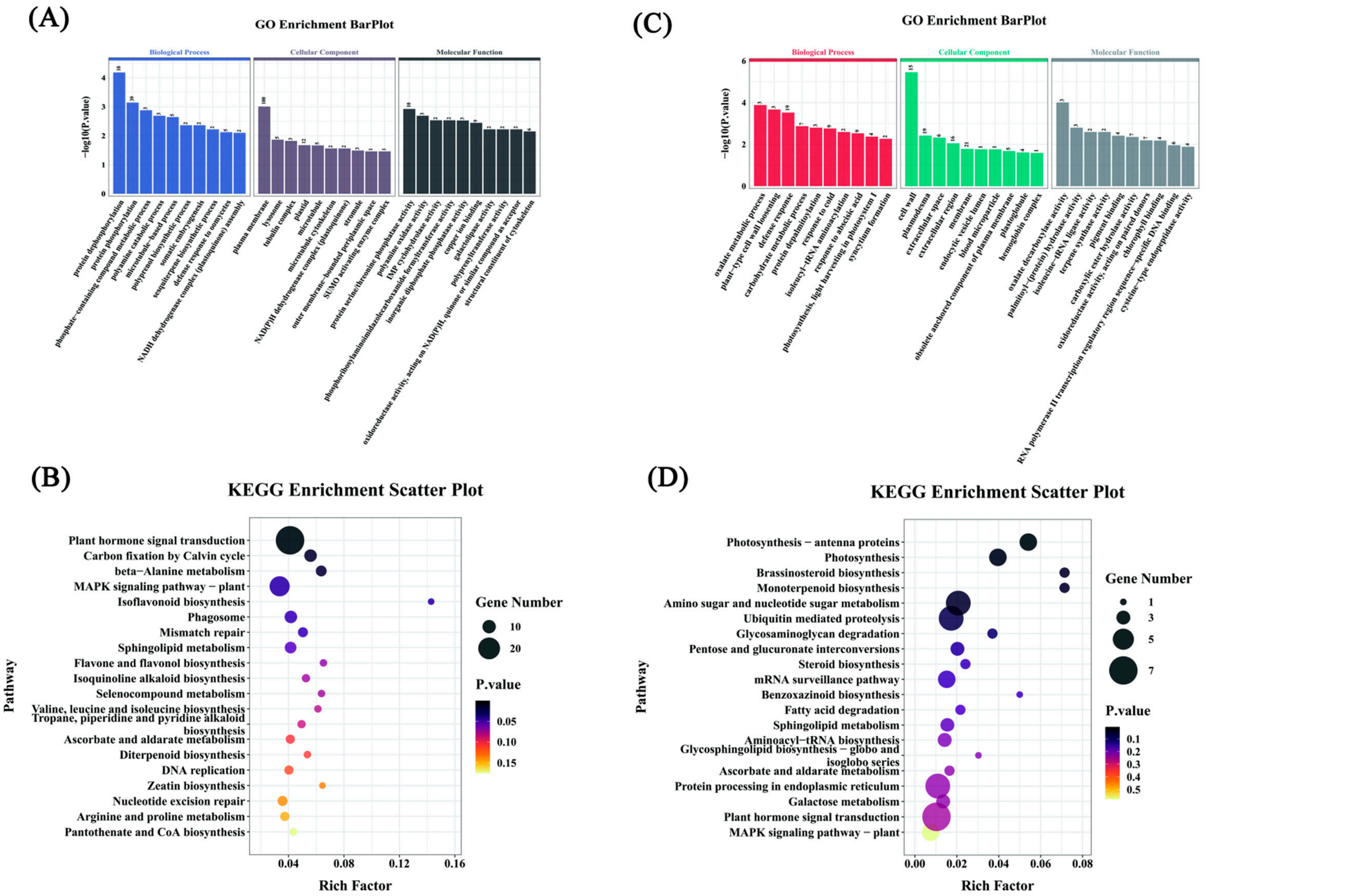

(A)
GO Enrichment BarPlot
Biological Process
Cellular Component
Molecular Function
−log10(P.value)
protein dephosphorylation
protein phosphorylation
phosphate−containing compound metabolic process
polyamine catabolic process
microtubule−based process
polyprenol biosynthetic process
somatic embryogenesis
sesquiterpene biosynthetic process
defense response to oomycetes
NADH dehydrogenase complex (plastoquinone) assembly
plasma membrane
lysosome
tubulin complex
plastid
microtubule
microtubule cytoskeleton
NAD(P)H dehydrogenase complex (plastoquinone)
stromule
outer membrane−bounded periplasmic space
SUMO activating enzyme complex
protein serine/threonine phosphatase activity
polyamine oxidase activity
IMP cyclohydrolase activity
phosphoribosylaminoimidazolecarboxamide formyltransferase activity
inorganic diphosphate phosphatase activity
copper ion binding
galactolipase activity
polyprenyltransferase activity
oxidoreductase activity, acting on NAD(P)H, quinone or similar compound as acceptor
structural constituent of cytoskeleton
(B)
KEGG Enrichment Scatter Plot
Pathway
Plant hormone signal transduction
Carbon fixation by Calvin cycle
beta−Alanine metabolism
MAPK signaling pathway − plant
Isoflavonoid biosynthesis
Phagosome
Mismatch repair
Sphingolipid metabolism
Flavone and flavonol biosynthesis
Isoquinoline alkaloid biosynthesis
Selenocompound metabolism
Valine, leucine and isoleucine biosynthesis
Tropane, piperidine and pyridine alkaloid biosynthesis
Ascorbate and aldarate metabolism
Diterpenoid biosynthesis
DNA replication
Zeatin biosynthesis
Nucleotide excision repair
Arginine and proline metabolism
Pantothenate and CoA biosynthesis
0.04 0.08 0.12 0.16
Rich Factor
Gene Number
10
20
P.value
0.05
0.10
0.15
(C)
GO Enrichment BarPlot
Biological Process
Cellular Component
Molecular Function
−log10(P.value)
oxalate metabolic process
plant−type cell wall loosening
defense response
carbohydrate metabolic process
protein depalmitoylation
response to cold
isoleucyl−tRNA aminoacylation
response to abscisic acid
photosynthesis, light harvesting in photosystem I
syncytium formation
cell wall
plasmodesma
extracellular space
extracellular region
membrane
endocytic vesicle lumen
blood microparticle
obsolete anchored component of plasma membrane
plastoglobule
hemoglobin complex
oxalate decarboxylase activity
palmitoyl−(protein) hydrolase activity
isoleucine−tRNA ligase activity
terpene synthase activity
pigment binding
carboxylic ester hydrolase activity
oxidoreductase activity, acting on paired donors
chlorophyll binding
RNA polymerase II transcription regulatory region sequence−specific DNA binding
cysteine−type endopeptidase activity
(D)
KEGG Enrichment Scatter Plot
Pathway
Photosynthesis − antenna proteins
Photosynthesis
Brassinosteroid biosynthesis
Monoterpenoid biosynthesis
Amino sugar and nucleotide sugar metabolism
Ubiquitin mediated proteolysis
Glycosaminoglycan degradation
Pentose and glucuronate interconversions
Steroid biosynthesis
mRNA surveillance pathway
Benzoxazinoid biosynthesis
Fatty acid degradation
Sphingolipid metabolism
Aminoacyl−tRNA biosynthesis
Glycosphingolipid biosynthesis − globo and isoglobo series
Ascorbate and aldarate metabolism
Protein processing in endoplasmic reticulum
Galactose metabolism
Plant hormone signal transduction
MAPK signaling pathway − plant
0.00 0.02 0.04 0.06 0.08
Rich Factor
Gene Number
1
3
5
7
P.value
0.1
0.2
0.3
0.4
0.5

Figure S2. GO and KEGG enrichment analysis of unique genes in tall fescue plants treated and untreated with silicon nanoparticles (Si NPs) under normal and drought stress conditions: (A) and (B) represent normal water conditions (CK vs Si NPs), and (C) and (D) represent drought conditions (DS vs DS_Si NPs). Treatment abbreviations: CK (control, well-watered), Si NPs (well-watered + 300 mg/L silicon nanoparticles), DS (drought stress), DS_Si NPs (drought stress + 300 mg/L silicon nanoparticles).

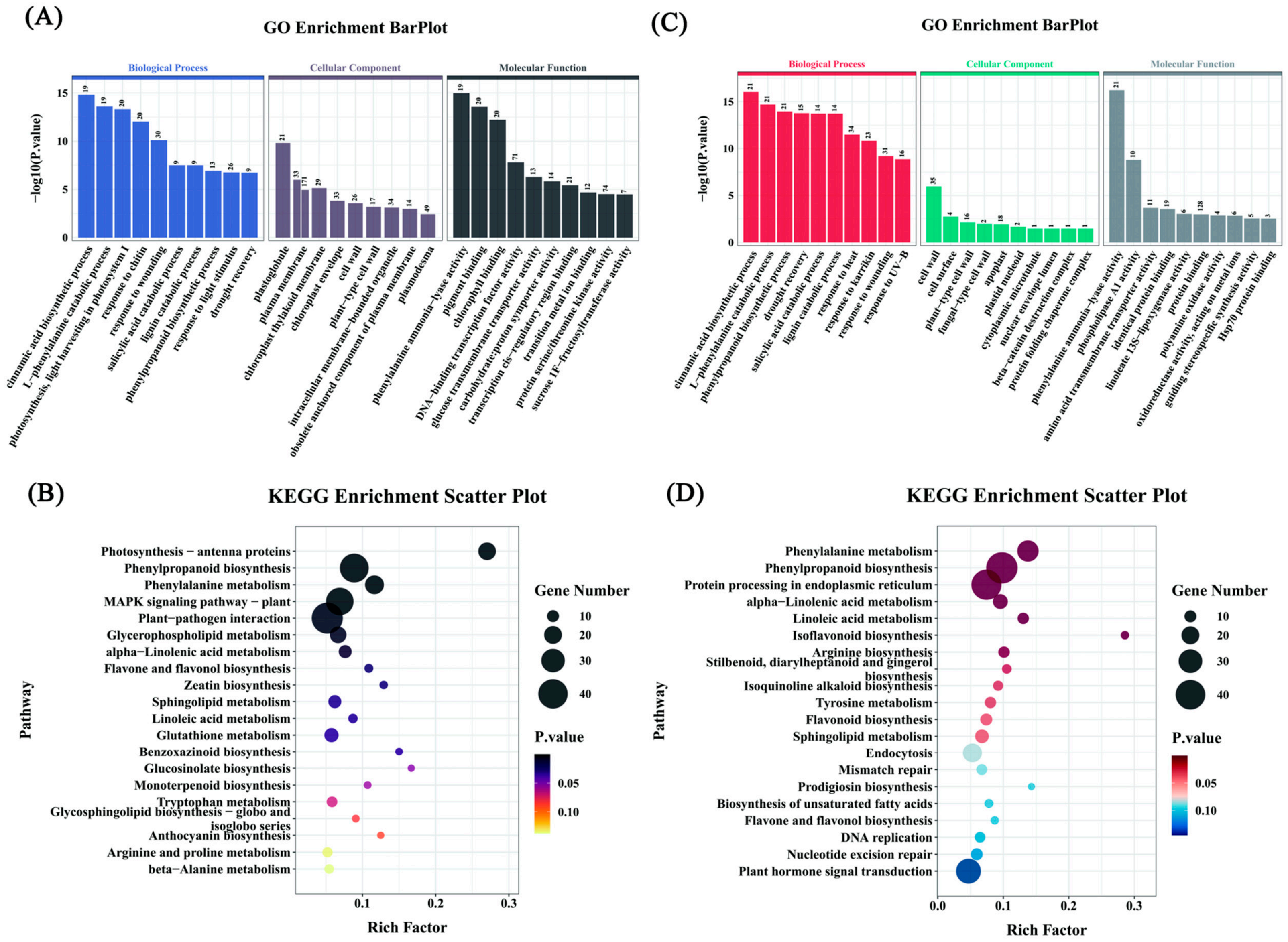
(A)
GO Enrichment BarPlot
Biological Process
Cellular Component
Molecular Function
−log10(P.value)
cinnamic acid biosynthetic process
L−phenylalanine catabolic process
photosynthesis, light harvesting in photosystem I
response to chitin
response to wounding
salicylic acid catabolic process
lignin catabolic process
phenylpropanoid biosynthetic process
response to light stimulus
drought recovery
plastoglobule
plasma membrane
chloroplast thylakoid membrane
chloroplast envelope
cell wall
plant−type cell wall
intracellular membrane−bounded organelle
obsolete anchored component of plasma membrane
plasmodesma
phenylalanine ammonia−lyase activity
pigment binding
chlorophyll binding
DNA−binding transcription factor activity
glucose transmembrane transporter activity
carbohydrate:proton symporter activity
transcription cis−regulatory region binding
transition metal ion binding
protein serine/threonine kinase activity
sucrose 1F−fructosyltransferase activity
(C)
GO Enrichment BarPlot
Biological Process
Cellular Component
Molecular Function
−log10(P.value)
cinnamic acid biosynthetic process
L−phenylalanine catabolic process
phenylpropanoid biosynthetic process
drought recovery
salicylic acid catabolic process
lignin catabolic process
response to heat
response to karrikin
response to wounding
response to UV−B
cell wall
cell surface
plant−type cell wall
fungal−type cell wall
apoplast
plastid nucleoid
cytoplasmic microtubule
nuclear envelope lumen
beta−catenin destruction complex
protein folding chaperone complex
phenylalanine ammonia−lyase activity
phospholipase A1 activity
amino acid transmembrane transporter activity
identical protein binding
linoleate 13S−lipoxygenase activity
protein binding
polyamine oxidase activity
oxidoreductase activity, acting on metal ions
guiding stereospecific synthesis activity
Hsp70 protein binding
(B)
KEGG Enrichment Scatter Plot
Pathway
Photosynthesis − antenna proteins
Phenylpropanoid biosynthesis
Phenylalanine metabolism
MAPK signaling pathway − plant
Plant−pathogen interaction
Glycerophospholipid metabolism
alpha−Linolenic acid metabolism
Flavone and flavonol biosynthesis
Zeatin biosynthesis
Sphingolipid metabolism
Linoleic acid metabolism
Glutathione metabolism
Benzoxazinoid biosynthesis
Glucosinolate biosynthesis
Monoterpenoid biosynthesis
Tryptophan metabolism
Glycosphingolipid biosynthesis − globo and isoglobo series
Anthocyanin biosynthesis
Arginine and proline metabolism
beta−Alanine metabolism
Rich Factor
Gene Number
P.value
(D)
KEGG Enrichment Scatter Plot
Pathway
Phenylalanine metabolism
Phenylpropanoid biosynthesis
Protein processing in endoplasmic reticulum
alpha−Linolenic acid metabolism
Linoleic acid metabolism
Isoflavonoid biosynthesis
Arginine biosynthesis
Stilbenoid, diarylheptanoid and gingerol biosynthesis
Isoquinoline alkaloid biosynthesis
Tyrosine metabolism
Flavonoid biosynthesis
Sphingolipid metabolism
Endocytosis
Mismatch repair
Prodigiosin biosynthesis
Biosynthesis of unsaturated fatty acids
Flavone and flavonol biosynthesis
DNA replication
Nucleotide excision repair
Plant hormone signal transduction
Rich Factor
Gene Number
P.value

Figure S3. GO and KEGG enrichment analysis of unique genes in tall fescue plants treated and untreated with cellulose nanocrystals (CNCs) under normal and drought stress conditions: (A) and (B) represent normal water conditions (CK vs CNCs), and (C) and (D) represent drought conditions (DS vs DS_CNCs). Treatment abbreviations: CK (control, well-watered), CNCs (well-watered + 100 mg/L cellulose nanocrystals), DS (drought stress), DS_CNCs (drought stress + 100 mg/L cellulose nanocrystals).

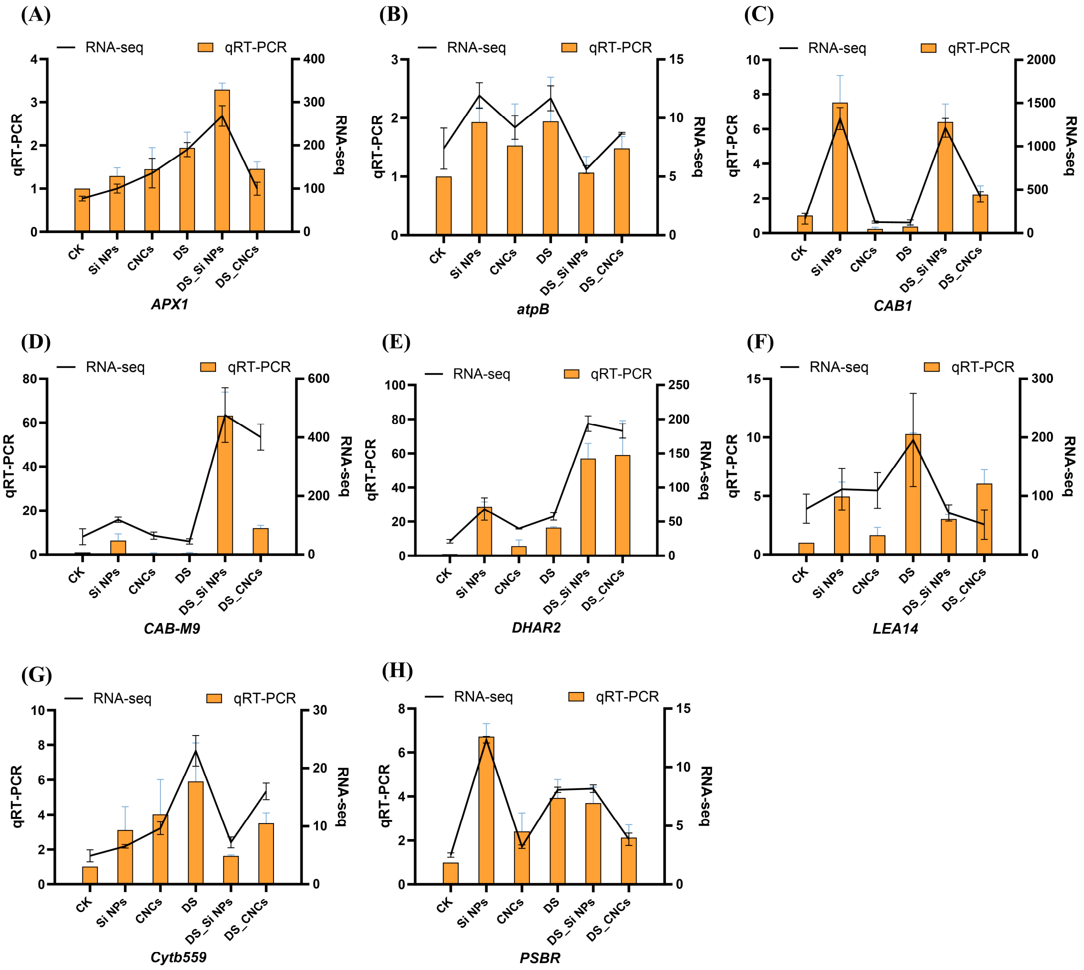

Figure S4. Validation results by qRT-PCR.

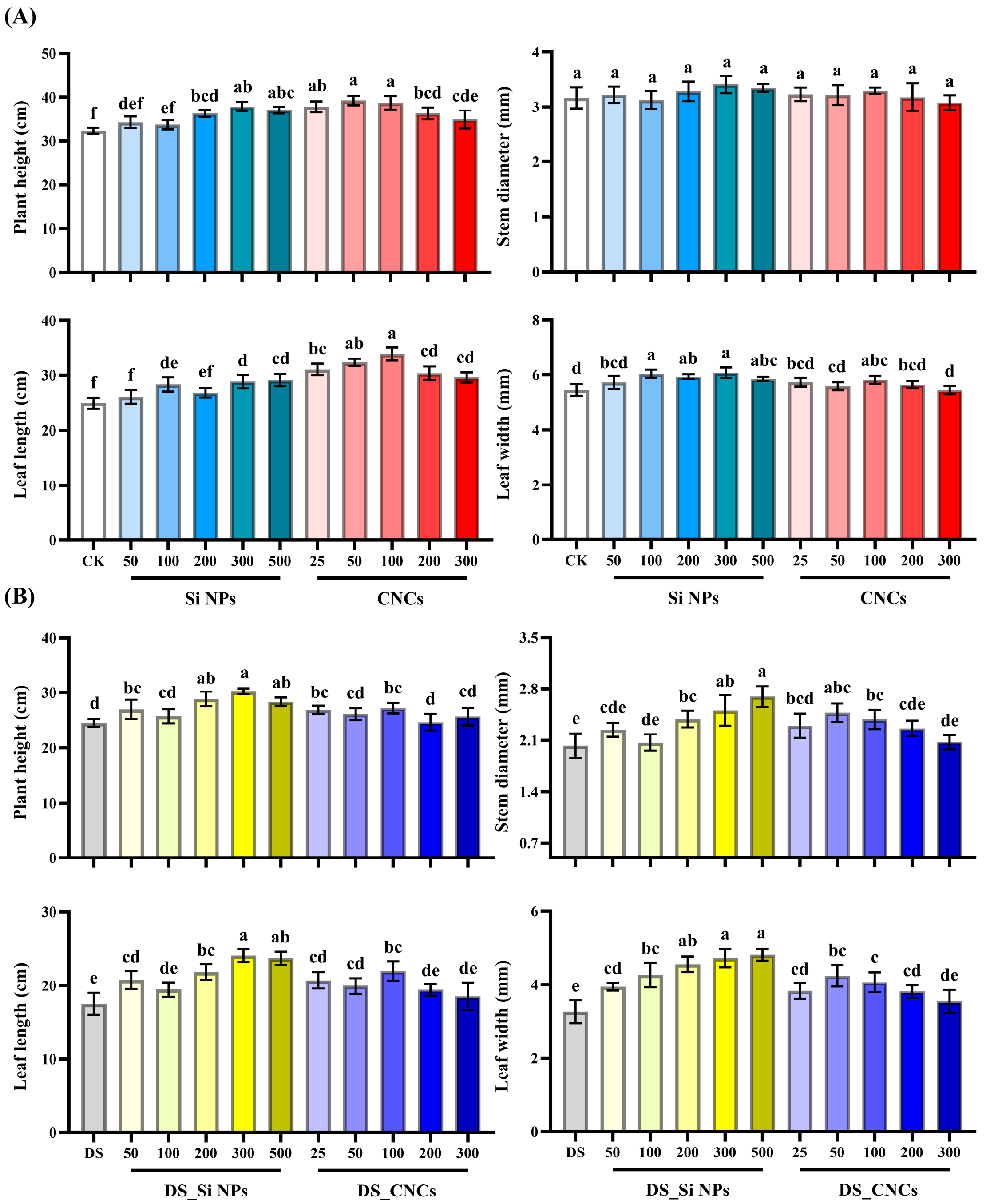

Figure S5. Effect of spraying different concentrations of silicon nanoparticles (Si NPs) and cellulose nanocrystals (CNCs) on the growth height, stem diameter, leaf length, and leaf width of tall fescue under normal (A) and drought (B) conditions. Data are presented as mean ± standard deviation from three biological experiments. Different letters above the bars indicate significant differences among treatments based on Tukey's HSD test (one-way ANOVA, $p < 0.05$). Treatment abbreviations: CK (control, well-watered), Si NPs (well-watered + 50, 100, 200, 300 and 500 mg/L silicon nanoparticles), CNCs (well-watered + 25, 50, 100, 200 and 300 mg/L cellulose nanocrystals), DS (drought stress), DS_Si NPs (drought stress + 50, 100, 200, 300 and 500 mg/L silicon nanoparticles), DS_CNCs (drought stress + 25, 50, 100, 200 and 300 mg/L cellulose nanocrystals).

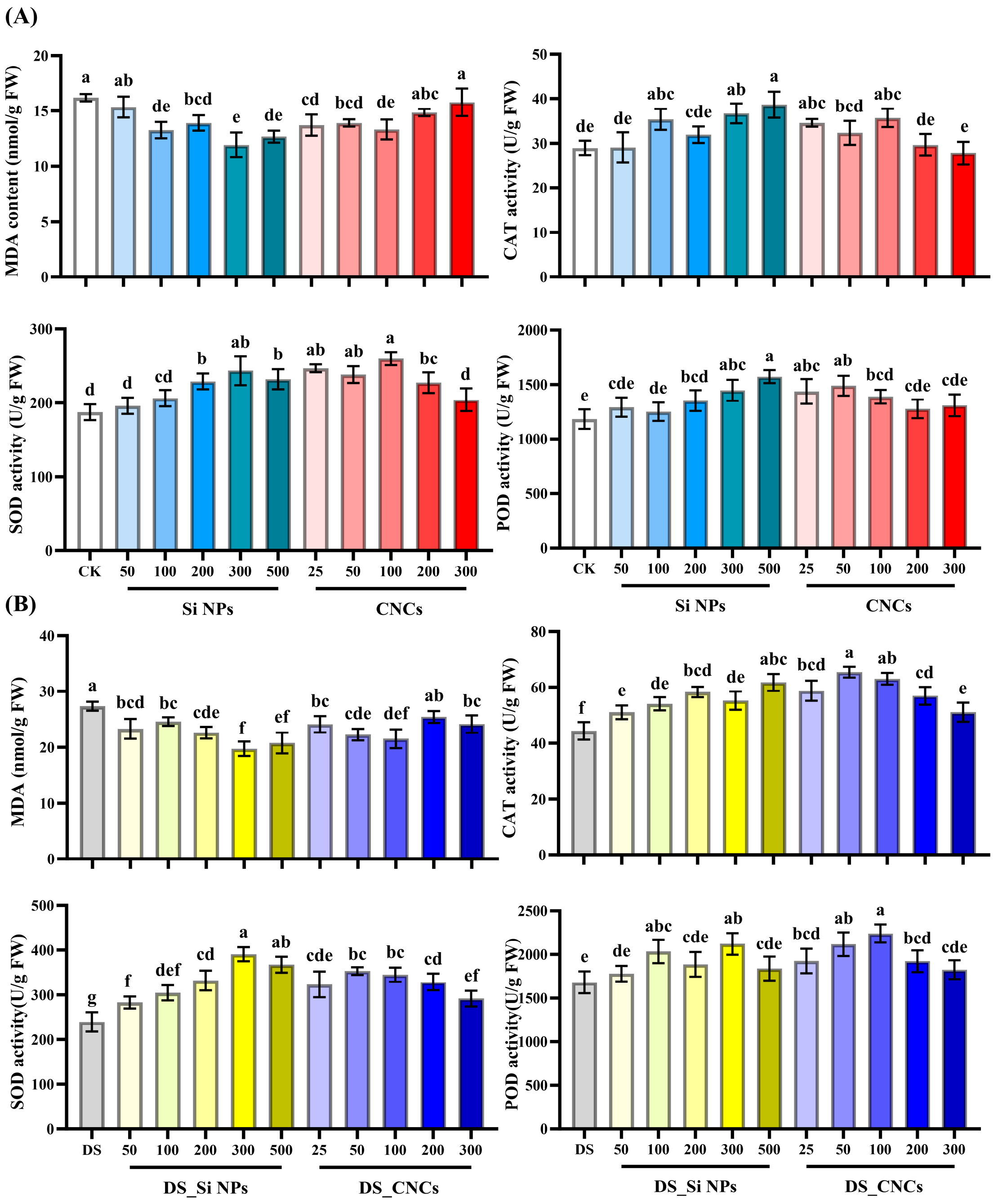

Figure S6. Effect of spraying different concentrations of silicon nanoparticles (Si NPs) and cellulose nanocrystals (CNCs) on the malondialdehyde (MDA) content and the activities of catalase (CAT), superoxide dismutase (SOD), and peroxidase (POD) in tall fescue under normal (A) and drought (B) conditions. Data are presented as mean ± standard deviation from three biological experiments. Different letters above the bars indicate significant differences among treatments based on Tukey's HSD test (one-way ANOVA, $p < 0.05$). Treatment abbreviations: CK (control, well-watered), Si NPs (well-watered + 50, 100, 200, 300 and 500 mg/L silicon nanoparticles), CNCs (well-watered + 25, 50, 100, 200 and 300 mg/L cellulose nanocrystals), DS (drought stress), DS_Si NPs (drought stress + 50, 100, 200, 300 and 500 mg/L silicon nanoparticles), DS_CNCs (drought stress + 25, 50, 100, 200 and 300 mg/L cellulose nanocrystals).

Table S1. Sequencing statistics for this study.

Sample	Clean reads	Clean bases	Valid%	Q20%	Q30%	GC%
CK_1	38358122	5.64G	96.61	98.23	94.55	54.86
CK_2	55511558	8.16G	96.32	98.13	94.33	53.75
CK_3	36479150	5.35G	95.48	97.88	93.60	54.02
CNCs_1	40340578	5.93G	97.08	98.05	94.26	54.13
CNCs_2	42402660	6.23G	96.27	98.34	95.05	54.08
CNCs_3	37181664	5.46G	96.05	97.82	93.39	54.25
Si NPs_1	45388724	6.66G	96.13	98.32	95.02	55.07
Si NPs_2	34367236	5.04G	95.69	98.12	94.16	54.19
Si NPs_3	34250114	5.02G	95.67	97.97	93.72	54.57
DS_1	38563140	5.66G	96.22	98.33	95.04	54.13
DS_2	38235832	5.61G	96.12	98.31	95.02	54.44
DS_3	44467488	6.52G	96.36	98.31	95.02	53.97
DS_CNCs_1	48040466	7.06G	96.72	98.24	94.78	54.13
DS_CNCs_2	35721012	5.24G	96.58	98.16	94.36	53.17
DS_CNCs_3	45811452	6.73G	96.65	98.28	94.94	54.66
DS_Si NPs_1	49229488	7.24G	96.77	98.12	94.26	54.68
DS_Si NPs_2	37861124	5.56G	95.91	98.09	94.12	54.5
DS_Si NPs_3	35298696	5.18G	96.47	98.08	94.12	54.54

Table S2. List of upregulated genes associated with nanomaterial addition.

Gene id	Symbol	Description	CK_TPM	CNCs_TPM	Si NPs_TPM	DS_TPM	DS_CNCs_TPM	DS_Si NPs_TPM	*P* -value	FDR
TRINITY_DN45747_c0_g2	*atpB*	*ATP synthase subunit beta, chloroplastic*	7.40	9.19	11.91	11.67	8.70	5.61	5.16635E-07	2.13102E-05
TRINITY_DN52275_c0_g2	*Cytb559*	*Cytochrome b-559 alpha subunit chloroplast*	4.94	9.65	6.55	22.99	16.01	7.23	0.006532834	0.045405233
TRINITY_DN109843_c0_g1	*PSBR*	*Photosystem II 10 kDa polypeptide, chloroplastic-like*	2.52	3.24	12.33	8.07	3.86	8.17	6.40606E-07	2.55532E-05
TRINITY_DN41235_c0_g2	*CAB-M9*	*Chlorophyll a-b binding protein of LHCII type 1*	60.70	64.00	118.47	44.70	400.99	475.71	1.72038E-07	1.31408E-05
TRINITY_DN34697_c0_g1	*CAB1*	*Light harvesting chlorophyll a/b-binding protein Lhcb1, partial*	164.07	125.67	1320.74	121.54	418.56	1214.76	1.20688E-06	6.28939E-05
TRINITY_DN121132_c0_g1	*DHAR2*	*Glutathione S-transferase DHAR2*	21.44	39.87	68.46	57.54	182.92	193.64	0.000118326	0.002298927
TRINITY_DN9236_c0_g1	*APX1*	*L-ascorbate peroxidase 1, cytosolic*	77.22	135.97	100.29	190.13	199.74	268.05	1.56046E-06	7.75845E-05
TRINITY_DN11	*GST3*	*Glutathione S-transferase*	0.86	6.03	20.51	1.07	21.11	29.33	0.005824	0.0427

3464_c0_g1									064	70009
TRINITY_DN51610_c0_g1	*CAT2*	*Catalase*	15.80	50.64	4.26	3.42	76.97	29.66	3.04498E-05	0.000816434
TRINITY_DN7007_c0_g1	*SAUR32-like*	*Small auxin-up RNA*	0.18	7.97	3.78	5.77	11.40	3.69	0.02893337	0.005810908
TRINITY_DN2790_c1_g1	*PIP2-7*	*Aquaporin PIP2-7*	8.46	2.56	5.87	0.94	11.50	13.05	7.61017E-06	0.000276633
TRINITY_DN37211_c0_g1	*DREBIB*	*Dehydration-responsive element-binding protein 1B*	0.82	4.60	16.28	1.99	26.29	18.79	0.000420134	0.006011032
TRINITY_DN14776_c0_g1	*SOD1*	*Superoxide dismutase [Cu-Zn]*	1.19	1.23	1.73	2.25	3.17	6.05	4.54611E-10	8.28048E-08
TRINITY_DN1598_c0_g1	*ERECTA*	*LRR receptor-like serine/threonine-protein kinase*	4.12	3.34	4.01	2.68	4.63	10.77	0.000301228	0.009429834
TRINITY_DN46192_c0_g1	*PRP*	*Pathogenesis-related protein*	0.14	1.39	1.60	0.06	2.41	3.86	0.000550587	0.014237731
TRINITY_DN16111_c0_g1	*BGLU5-like*	*Beta-glucosidase 5-like*	43.39	33.40	83.92	3.07	55.98	15.86	0.000183064	0.003234869

TRINITY_DN7634_c0_g1	*CCR*	*Cinnamoyl CoA reductase*	5.08	3.97	1.79	1.94	7.52	5.82	0.000488527	0.006759882
TRINITY_DN20900_c0_g1	*LSI2*	*Silicon efflux transporter*	0.99	0.65	2.51	2.72	2.71	6.33	0.000192341	0.003365832

Table S3. Primers used in qRT-PCR.

Gene ID	Primer	Sequence (5′-3′)
APX1	Sense	AGGTGCCACAAGGAGAGGTCTG
	Anti-sense	CAGGGTCAGTCAGCAGGGTTTTG
DHAR2	Sense	ACCGTGGAGGTGCTGGTCAAG
	Anti-sense	TTGGTCTGGTAGGGCTCCTTCTTC
PSBR	Sense	AGGTCCAGACTACTCAGCCTTTCG
	Anti-sense	GGTAGACGCCCTTCCCCTTGG
Cytb559	Sense	GTCTGGAAGCACGGGAGAACG
	Anti-sense	GGCCGAGGACTTCCAAACACG
atpB	Sense	GCTATGAGTGCTACAGACGGGTTG
	Anti-sense	ATTGTCAACAGGCTCCCCAAGAAC
CAB1	Sense	TCGTCCACGCCCAGAGCATC
	Anti-sense	CGAAGCTGCCTCCGGGGTAG
CAB-M9	Sense	GAGACCTTTGCCAAGAACCGTGAG
	Anti-sense	AGGGAAGACGCAGCCGAGAG
LEA14	Sense	CAAGGAGTTCGTGGCGGACAAG
	Anti-sense	GGGATGCGGTGGGAGTAGGG
